# Survey of Pesticide Residues in Vegetables in the Albanian Market and Associated Dietary Exposure

**DOI:** 10.3390/foods15101761

**Published:** 2026-05-15

**Authors:** Elda Marku, Matilda Likaj, Ridvana Mediu, Jonida Tahiraj, Sonila Shehu, Aurel Nuro, Vjollca Vladi

**Affiliations:** 1 Faculty of Natural Sciences, University of Tirana, 1000 Tirana, Albania; 2 Faculty of Applied Science, University College LOGOS, 1000 Tirana, Albania; 3 Food Safety and Veterinary Institute, 1000 Tirana, Albania

**Keywords:** pesticide, multi-residue analysis, chromatography-tandem mass spectrometry, vegetables, dietary exposure, maximum residue limits, acceptable daily intake, Albania

## Abstract

Vegetables constitute an essential component of the daily diet in Albania; however, they also represent a major pathway of human exposure to pesticide residues. This study investigates the presence of pesticide residues in widely used vegetables, including leafy, fruity, root, and bulb types, and evaluates the potential dietary health risks associated with their consumption. Vegetable samples were analyzed using gas chromatography–tandem mass spectrometry (GC-MS/MS) and liquid chromatography–tandem mass spectrometry (LC-MS/MS), for the presence of 417 pesticide analytes, ensuring high analytical sensitivity and reliability. Pesticide residues were present, with 42 distinct compounds, including metabolites, found in all the analyzed samples. Notably, some of the detected substances are not currently authorized for use as plant protection products, suggesting either environmental persistence or regulatory non-compliance. Exceedances of European Union maximum residue limits (MRLs) were most frequently detected in leafy vegetables (42.31%), followed by fruity vegetables (18.75%), whereas no MRL exceedances were observed in root and bulb vegetables. According to the dietary exposure assessment conducted using European Food Safety Authority Pesticide Residue Intake Model (EFSA PRIMo model v.3.1), chronic dietary exposure to pesticide residues was below the acceptable daily intake (ADI). According to this assessment, the acute exposure exceeded the acute reference dose (ARfD) for several pesticide–vegetable combinations, particularly among children. This highlights the need for ongoing monitoring and better agricultural management techniques to reduce potential health risks related to pesticide residues in vegetables. The study results indicate the need to strengthen national monitoring programs, enforce pesticide regulations more strictly, and promote the wider adoption of integrated pest management strategies to reduce dietary pesticide exposure and protect public health in Albania.

## 1. Introduction

Vegetables are an essential part of a balanced diet, providing nutrients that prevent deficiencies and lower the risk of chronic illnesses [1]. Beyond basic nutrition, they contain bioactive compounds that contribute to important physiological processes and overall health [2]. Vegetables are a key part of Mediterranean diet and contribute substantially to daily food consumption [3]. In recent years, the demand for leafy vegetables has increased dietary modifications to encourage healthier living [4].

In 2024, Albania cultivated 34,696 hectares of vegetables. The total production reached 1.42 million tons, which is 2.9% higher than in 2023. About two-thirds of these outputs were fresh vegetables such as tomatoes, cucumbers, and peppers, with greenhouse production accounting for nearly 25% [5,6].

Pesticide use in agriculture has raised concerns about the safety of locally grown vegetables. Pesticide residues can remain on produce for extended periods. Their potential health effects depend on both the concentration and toxicity of the chemicals. Contamination levels differ between vegetable types and are shaped by farming practices, the chemical nature of the pesticides, and post-harvest handling [7]. Chemical pesticides are applied in agriculture to prevent damage from pests, diseases, fungi, rodents, and weeds [8]. People are mainly exposed to pesticides through the consumption of contaminated produce [9,10]. The European Food Safety Authority (EFSA) annually evaluates pesticide residues in food across EU member states. According to the latest report, more than 146,000 food samples were analysed in 2023, with consumer exposure generally considered low. The most frequently detected residues in fruits and vegetables included boscalid, fludioxonil, cyprodinil, pyrimethanil, azoxystrobin and acetamiprid. These residues were commonly reported in peppers, tomatoes, lettuce, cucumbers, grapes, berries and leafy vegetables across the European market [11]. Different pesticide residues were found in the vegetable samples collected from Slovenian stores, including boscalid, fluopyram, pyraclostrobin and tebuconazole [12]. Recent studies of vegetable samples from Polish production have reported the presence of pesticide residues, including non-approved ones such as chlorpyrifos. Several of these samples were multi-residue [13,14]. Pesticide compounds have been reported in vegetable samples in Sicily, Italy [15]. Similar findings of multi-residue contamination of tomato samples have also been reported in Western Sardinia, Italy [16].

EU regulations have set maximum residue limits (MRLs) of pesticides in food [17]. Pesticide residues have been detected in exported food from Balkan countries to EU. Greece, Albania and Serbia, were identified as the most problematic countries [18], having the highest number of pesticide residues and notifications. There is a lack of recent research data about the pesticide residues in vegetables in Albania. The National Food Authority does not make the monitoring data publicly available. Several official reports in Albania have reported pesticide residues above these threshold levels in fruits and vegetables [19,20]. Recent studies have revealed multi-residue contamination in Albanian crops, underscoring the need for continuous monitoring and stricter regulatory enforcement [21,22]. To our knowledge, this is the first study to investigate the presence of 417 pesticide residues in a wide range of vegetable types intended for domestic production in Albania, from different cultivation methods, and to provide a risk assessment analysis.

Monitoring pesticide residues in food was carried out using different analytical methods. Complex and time-consuming sample preparation methods have been replaced by the QuEChERS method, which is valued for its simplicity, cost-effectiveness, and low solvent consumption [23].

This study investigates the occurrence of various pesticide residues in the frequently consumed vegetables, such as leafy, fruity, root, and bulb categories. The collected vegetable samples were intended for the Albanian market. Analysis was performed using QuEChERS approach according to EN 15662, a widely applied European standardized analytical method for multi-residue analysis in food matrices, followed by LC-MS/MS and GC-MS/MS techniques. Potential dietary risks from pesticide residues were assessed through the comparison of chronic and acute exposure estimates with toxicological reference values ADI and ARfD, providing information which helps safeguard public health and strengthen pesticide monitoring control.

## 2. Materials and Methods

### 2.1. Study Area and Sampling

To achieve comprehensive geographical coverage, samples were obtained from several regions throughout Albania, with priority given to areas characterized by higher production levels, based on data provided by the Institute of Statistics (INSTAT), and targeting the most locally produced vegetables. The sampling sites included the following regions: Shkoder, Puka, Kukes, Elbasan, Tirana, Durres, Lushnje, Divjake, Fier, Berat, Korça, ensuring representation of diverse agro-climatic conditions and farming practices.

A structured sampling approach was applied, considering both crop type (fruity, leafy, root, and bulb vegetables) and production system (greenhouse versus open-field cultivation). The selected vegetables included types that constitute a significant proportion of the daily diet in the Albanian population. Vegetables cultivated in greenhouses were sampled at different time points throughout the production season, whereas open-field vegetables were collected during their respective harvest periods, in accordance with the harvesting schedules of each crop. Sampling design details, including region, cultivation method, vegetable type, and planting and harvesting periods are provided in Supplementary Materials. In all cases, sampling was conducted on the day of harvest immediately before marketing.

Sampling was carried out in accordance with the methodologies approved by the Ministry of Agriculture and Rural Development (Order No. 253, 27 August 2009) and the European Commission Directive 2002/63/EC, [24]. A total of 62 samples were collected from 11 different types of vegetables, as presented in Table 1. Samples had 26 leafy vegetables (i.e., lettuce, spinach, parsley, dill, arugula), 17 fruity vegetables represented from peppers, 11 root vegetables including potatoes and carrots, and 8 bulb vegetables, onions and leeks.

### 2.2. Chemicals and Standards

Pesticide analytical standards comprising 197 analytes for GC-MS/MS and 220 analytes for LC-MS/MS were used in this study. GC pesticide standard mixtures were obtained from Restek (Bellefonte, PA, USA) and included compounds from several pesticide classes, such as organophosphorus compounds, organochlorines, synthetic pyrethroids, herbicide methyl esters, and organonitrogen compounds. LC pesticide standard mixtures were obtained from Agilent Technologies (Santa Clara, CA, USA). Matrix-matched calibration standards were prepared at concentrations of 0.01, 0.02, 0.05, 0.1, and 0.2 mg/kg. All LC- and GC-grade solvents were purchased from VWR (Radnor, PA, USA), while QuEChERS extraction kits were obtained from Chromabond (Düren, Germany).

### 2.3. Sample Preparation and Extraction

Vegetable samples were homogenized upon arrival at the laboratory. The samples not analyzed immediately were stored in −200 °C. 10 g of the homogenized sample was weighed in a centrifuge tube. Sample preparation was performed using the QuEChERS approach according to the EN 15662 standard [25]. Pesticide residues were quantified using gas chromatography–tandem mass spectrometry (GC-MS/MS) and liquid chromatography–tandem mass spectrometry (LC-MS/MS) following acetonitrile extraction and PSA-based clean-up.

The extraction was initiated by adding 10 mL of acetonitrile to 10 g of homogenized sample. The CHROMABOND QuEChERS Mix I kit [23] was added, containing 4 g MgSO_4_, 1 g NaCl, 500 mg Disodium Hydrogen Citrate Sesquihydrate (Na_2_H-citrate × 1.5 H_2_O), and 1 g Trisodium Citrate Dihydrate (Na_3_C_6_H_5_O_7_·2H_2_O). After centrifugation, 6 mL of the resulting aliquot of the organic phase was subsequently purified using CHROMABOND QuEChERS Clean-up Mix III containing 150 mg PSA and 900 mg anhydrous MgSO_4_. The final extract was directly injected into LC-MS/MS and GC-MS/MS instruments for the quantification of pesticide residues.

### 2.4. Instrumental Analysis

#### 2.4.1. Instrumentation

LC–MS/MS analysis was performed utilizing the Agilent Infinity 1260 system coupled to Agilent 6460 triple quadrupole mass spectrometry detector (Agilent Technologies, Inc., Santa Clara, CA, USA). The separation of the analytes was carried out on a C18 column (1.8 µm 150 × 2.1 mm), at ambient temperature. For the LC-MS/MS analysis, a binary mobile phase consisting of (A) ultrapure water with 5 mM ammonium formate and 0.1% formic acid and (B) methanol with 5 mM ammonium formate and 0.1% formic acid was used. Chromatographic separation was performed using gradient elution at a constant flow rate of 0.400 mL/min. The initial composition (95% A, 5% B at 0.50 min) was linearly increased to 40% B at 3.00 min and further to 100% B at 17.00 min, held until 19.01 min, and then returned to initial conditions (5% B) at 19.10 min for column re-equilibration. An electrospray ionisation (ESI) source operating in both positive and negative modes was used for analyte ionisation. The volatile compounds were analyzed using an Agilent 7890B gas chromatograph (Agilent Technologies, Inc., Santa Clara, CA, USA) coupled to an Agilent 7000D triple quadrupole mass spectrometer, using a capillary electron impact (EI) source. Separation was performed on an Agilent DB-5ms Ultra Inert column (30 m × 0.25 mm × 0.25 μm) with 1 μL pulsed splitless injection. The oven was programmed from 70 °C to 340 °C. Injector, transfer line, and source temperatures were 260 °C, 330 °C, and 250 °C, respectively. Carrier gas flow was 1.1 mL/min. Total run time: 41 min. To ensure precise quantitation of analytes, both techniques were operated in multiple reactions monitoring (MRM) transitions mode for each target pesticide. The optimized acquisition parameters are provided in the Supplementary Materials.

#### 2.4.2. Method Validation

A multi-residue analytical method was used for the determination of pesticide residues in vegetable samples. The method was validated in accordance with the requirements specified in SANTE 11312/2021 [26,27] and ISO/IEC 17025:2017 [28]. Validity of the method was determined by the fulfilment of the parameters linearity, trueness, and precision, limit of detection (LOD) and limit of quantification (LOQ), as defined in the SANTE guideline.

The recovery experiments were performed at a spiking level of 0.01 mg/kg, which corresponds to the method’s LOQ. Linearity was assessed by analyzing five calibration solutions prepared in the sample matrix. Duplicate injections for each concentration were performed at the beginning and end of the analytical sequence to monitor instrument performance in the range of 0.01–0.2 μg/kg. The correlation coefficients (r^2^) for all analytes exceeded 0.98. Fortified samples at the LOQ level (0.01 μg/kg) were used to assess accuracy (trueness), and the results met the SANTE acceptability range of 70–120%. The method validation parameters are provided in the Supplementary Materials. The method’s performance was confirmed using both LC-MS/MS and GC-MS/MS techniques.

### 2.5. Dietary Exposure and Risk Assessment

The dietary exposure assessment for pesticide residues was conducted using the European Food Safety Authority (EFSA) Pesticide Residue Intake Model (PRIMo), revision 3.1 [29,30,31]. This model uses these elements: age groups and data on food consumption, MRL, supervised trials median residue for raw agricultural commodity (STMR) in mg/kg, and the highest residue (HR) found in samples in mg/kg. The exposure calculation was performed within the post-marketing scenarios, in which values of HR and STMR were replaced with residue concentration values measured in the analyzed samples. Mean residue concentrations were used to represent typical exposure conditions, while maximum detected levels were included to account for a conservative (worst-case) scenario. Despite the inherent limitations of the available data, this approach ensures a reasonable and sufficiently protective evaluation of consumer risk. Values below the LOQ were not included in the risk assessment. Due to the lack of nationally representative consumption data for Albania, dietary intake data from Italy were used as an approximation, providing average values as a single reference point and not allowing the differentiation of low and high consumption scenarios.

The assumption is based on the similarities between Albanian and Italian dietary patterns. Both countries have a Mediterranean-type diet with a high consumption of vegetables. In the absence of nationally representative data for food consumption in Albania, the use of proxy data is a common practice in exposure assessments; however, differences in dietary patterns between populations may influence the accuracy of the estimates [32,33]. Consequently, this represents a methodological constraint, and the resultant exposure estimates should be regarded as indicative rather than fully representative of the Albanian population. Both long-term (chronic) and short-term (acute) exposure were calculated for adults and toddlers.

#### 2.5.1. Assessment of Acute Exposure

The acute exposure is estimated using the International Estimated Short-Term Intake (IESTI, mg/kg bw) and compared to the Acute Reference Dose (ARfD, mg/kg bw), which represents the amount of an active substance that can be consumed in a short period without posing a health risk [29], as follows:

$$IESTI=\frac{HR\times LP\times PF\times CF}{BW}$$

where:

HR is the highest residue found in samples (mg/kg);
LP is the highest consumption level for each age group (g/kg bw);
PF is the processing factor (=1 for raw commodities);
CF is the residue conversion factor for risk assessment.
BW is the body weight (kg).

The percentage of the Acute Reference Dose (%ARfD) was calculated to show the proportion of the safe one-day pesticide dose consumed and to estimate whether acute exposure was acceptable. Acute risk was considered acceptable when the exposure value was below 100% of the ARfD [33,34,35].

#### 2.5.2. Assessment of Chronic Exposure

Chronic exposure is estimated using the National Estimated Daily Intake (NEDI, mg/kg bw/day) and compared with the Acceptable Daily Intake (ADI), which is the amount of the pesticide that can be consumed daily without adverse effects [29].

$$NEDI=\frac{APR\times average\;daily\;consumption}{1000}$$

where APR is the mean concentration of pesticide residues in analyzed samples (mg/kg).

%ADI = NEDI × 100/ADI(1)

Chronic risk is acceptable when the exposure value is below 100% of the ADI [35,36].

## 3. Results and Discussion

The concentrations of pesticide residues found in leafy, fruity, bulb, and root vegetables gathered from various regions of Albania are presented herein to enable comparison of the levels and occurrence of residues among the different vegetable groups. Table 2, Table 3, Table 4 and Table 5 provide a summary of the relevant data.

**Figure 1:**
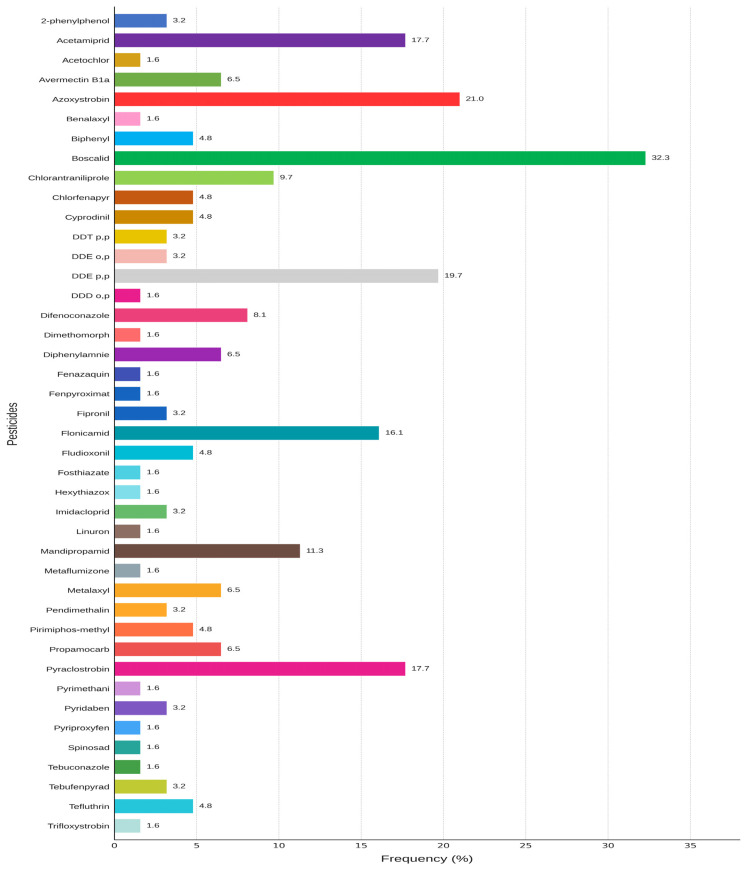
Detection frequency of identified pesticides.

The data presented above demonstrate that leafy vegetables had the highest frequency of MRL exceedances among the investigated vegetable groups. Some vegetables contained residues of up to ten different pesticides, reflecting intensive crop protection practices involving repeated or overlapping treatments during the growing season. This is possibly driven by persistent pest and disease challenges. The occurrence of multiple pesticide residues (≥2) was predominant being observed in 56.45% of samples. 22.58% of samples contained a single residue, while 20.97% of them contained no detectable pesticide residue. Of particular concern is the presence of multiple pesticides in the same sample exceeding the legally established limits. The analyzed samples contained 42 different pesticides, including their metabolites, and the detection frequency of each compound is presented in Figure 1.

The occurrence of pesticide residues across the analyzed commodities is summarized in Table 6. Pesticides with higher detected frequencies were boscalid (32.3%), followed by azoxystrobin (21.0%), indicating frequent fungicide applications driven by strong fungal pressure. The detection of p,p′-DDE (19.7%) points to residual environmental persistence from intensive past use of DDTs in Albania. Acetamiprid and pyraclostrobin also exhibited relatively high occurrence (17.7%), along with flonicamid (16.1%), mandipropamid (11.3%), chlorantraniliprole (9.7%), and difenoconazole (8.1%).

The frequent presence of fungicides in vegetables has been reported in different studies conducted in European countries, such as Spain [37], Italy [16,38], Switzerland [39], Germany [40], and Cyprus [41]. According to these studies, non-compliant samples for pesticide residues with EU origin have also been identified in the domestic market.

For each commodity, the most frequently detected analytes, along with their detection frequency, mean concentration, and maximum concentrations, are reported in Table 6.

**Figure 2:**
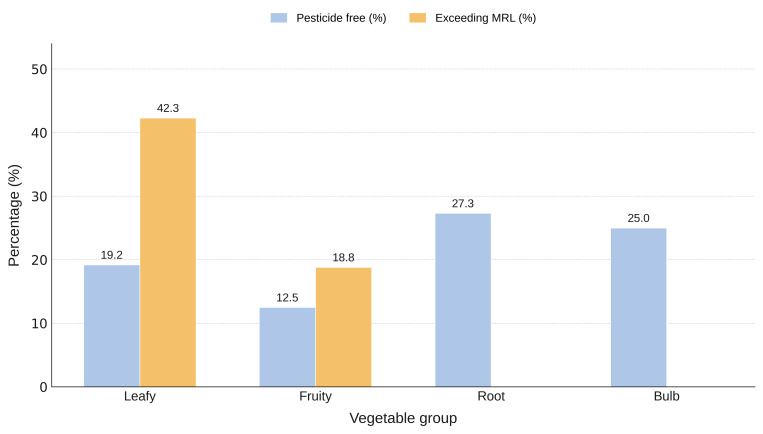
Distribution of pesticide-free samples and MRL exceedances by vegetable group.

The distribution of pesticide-free samples and MRL exceedances by vegetable group is presented in Figure 2.

Leafy green vegetables showed the highest rate of MRL exceedances, with 11 of 26 samples (42.31%) classified as non-compliant. This may be attributed to their rapid growth cycle and short pre-harvest periods that limit residue degradation. 18.75% of the fruity vegetables were non-compliant samples (3 of 16 samples), whereas no exceedances were detected in the bulb and root vegetables. Among the pesticides detected in the analyzed samples, 11 are not approved to be used as plant protection products, such as Acetochlor, Benalaxyl, Biphenyl, Chlorfenapyr, DDT, Dimethomorph, Diphenylamine, Fipronil, Imidacloprid, Linuron and Metaflumizone. The identified pesticides were classified according to their primary use, hazard classification, and chemical class [42]. A summary of this categorization is presented in Table 7.

Leafy green vegetables showed the highest rate of MRL exceedances, with 11 of 26 samples (42.31%) classified as non-compliant, which may be attributed to their rapid growth cycle and short pre-harvest periods that limit residue degradation. Fruit vegetables followed with 18.75%, whereas no exceedances were detected in bulb and root vegetables.

Fungicides and insecticides were the most commonly found pesticide classes, indicating that pest and disease management is mainly focused on controlling fungal and insect pressures. Moderate-risk pesticides constituted the predominant category (36.6%), while slight-risk and high-risk chemicals represented 19.5% and 7.3%, respectively.

The statistical analysis of analytical data related to the cultivation system was conducted using Minitab (version 18.1, Minitab Inc., Herndon, VA, USA). First, the data distribution was evaluated via the Anderson–Darling normality test and probability plots. The results revealed that neither group’s data followed a normal distribution (*p* < 0.05), and therefore, non-parametric statistical methods were applied. Total pesticide residue concentrations were compared by performing the Kruskal–Wallis test for two cultivation systems (greenhouse versus open field), as well as for two commodity types (leafy and fruity vegetables).

For leafy vegetables, the Kruskal–Wallis test revealed a statistically significant difference in total pesticide residue concentrations between cultivation systems (H = 11.70, df = 1, *p* = 0.001). Greenhouse samples exhibited substantially higher median residue levels (3.82 mg/kg) compared to open-field samples (0.0055 mg/kg). The higher mean rank observed for greenhouse samples (17.5 vs. 7.0) further confirms the elevated pesticide contamination under greenhouse conditions.

For fruity vegetables, the Kruskal–Wallis test indicated a significant variation in total pesticide residue levels across different cultivation methods (H = 10.62, df = 1, *p* = 0.001). The median residue concentrations in greenhouse samples were notably higher (0.13 mg/kg) than those found in open-field samples. Additionally, the mean rank for greenhouse samples was greater (11.9 compared to 3.7), reinforcing the conclusion of increased pesticide contamination within greenhouse environments. A statistically significant difference was observed, with greenhouse fruiting samples showing higher pesticide residue levels. Leafy vegetables exhibited significantly higher pesticide residue levels compared to fruity vegetables.

A Kruskal–Wallis test revealed a statistically significant difference in total pesticide residue concentrations between leafy and fruity vegetables (H = 4.43, df = 1, *p* = 0.035), with leafy vegetables exhibiting higher median residue levels (0.3095 mg/kg) compared to fruity vegetables (0.0840 mg/kg), suggesting a greater tendency for pesticide accumulation in this commodity group. For fruity vegetables, the Kruskal–Wallis test indicated a significant variation in total pesticide residue levels across different cultivation methods (H = 10.62, df = 1, *p* = 0.001). The median residue concentrations in greenhouse samples were notably higher (0.13 mg/kg) than those found in open-field samples. Additionally, the mean rank for greenhouse samples was greater (11.9 compared to 3.7), reinforcing the conclusion of increased pesticide contamination within greenhouse environments. A statistically significant difference was observed, with greenhouse fruiting samples showing higher pesticide residue levels. Leafy vegetables exhibited significantly higher pesticide residue levels compared to fruity vegetables. The statistical analysis is provided in the Supplementary Materials.

To evaluate the association between cultivation method and the occurrence of multiple pesticide residues, the Chi-square test of independence was used. The Chi-square test of independence was performed on the combined dataset (including both leafy and fruity vegetables). It revealed a highly significant association between cultivation method and the occurrence of multiple pesticide residues (χ^2^ = 20.90, df = 1, *p* < 0.001). Greenhouse samples showed a substantially higher frequency of multiple residues compared to open-field samples. The analysis indicates that greenhouse cultivation is strongly associated with an increased likelihood of multiple pesticide contamination, regardless of vegetable type. The consistent findings observed across individual commodity groups and the combined dataset provide evidence that cultivation method influences both pesticide residue concentrations and the occurrence of multiple residues. In particular, greenhouse cultivation was associated with elevated residue levels and a higher occurrence of multiple pesticide contamination, especially in leafy vegetables.

The higher number of non-compliant samples observed in greenhouse production compared to open-field cultivation suggests a significant influence of microclimatic conditions on pesticide residue levels. As shown in Table 8, cities characterized by cooler climates, such as Shkodra, Korça, Puka, and Kukës show a higher proportion of samples below the MRL or pesticide-free. In contrast, greenhouse systems recorded the greatest number of alert samples and the lowest proportion of pesticide-free products. This pattern can be attributed to specific environmental conditions in greenhouses, particularly elevated temperatures and high relative humidity, which may increase pest and disease pressure, accelerate pest life cycles, and consequently raise the frequency and intensity of pesticide applications. Additionally, limited air circulation and reduced exposure to natural degradation could slow pesticide dissipation, resulting in higher detectable residue levels. These findings indicate that greenhouse residue profiles are strongly influenced by controlled microclimatic parameters. Optimizing temperature regulation, humidity management, and ventilation systems in greenhouses to approximate conditions found in cooler northern and southeastern regions of Albania may reduce residue accumulation and improve compliance with food safety standards.

**Figure 3:**
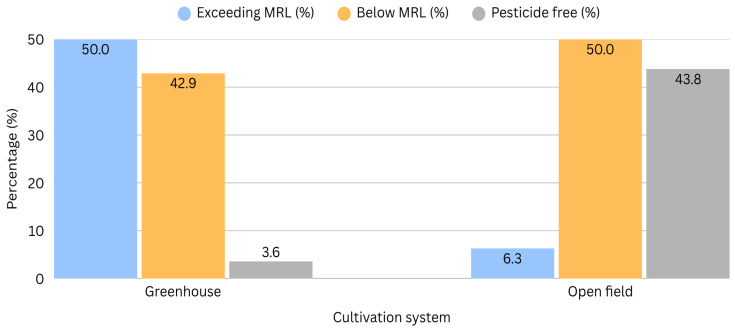
The distribution of pesticide residues in greenhouses and open fields.

This comparison was made only for leafy and fruity vegetables sampled from both open-field and greenhouse systems. A schematic representation of the results is provided in Figure 3. The root and bulb vegetables were collected exclusively from open-field cultivation and were therefore excluded from this table.

### Dietary Risk Assessment

Chronic dietary exposure (%ADI) was estimated using the PRIMo model v3.1, only for vegetables included in the model consumption database, as shown in Table 9. Analytes detected below the limit of quantification (LOQ) were not considered in the exposure assessment.

The calculated exposure levels represented only a small fraction of the ADI, indicating that the long-term dietary intake of these pesticide residues is unlikely to pose a risk to consumer health.

The acute dietary exposure data were calculated using ARfD in Primo v3.1, based on the large portion consumption of the most sensitive consumer group (Table 10).

The acute dietary exposure (%ARfD) exceeded the reference values for children for several pesticide–vegetable combinations, including lettuce (Pyraclostrobin 369%, Tebuconazole 310%), spinach (Pyraclostrobin 118%, Tebuconazole 153%), and peppers (Acetamiprid 321%). For adults, exceedances were observed for lettuce (Pyraclostrobin 118%, Tebuconazole nearly 99%), highlighting a potential acute health risk for the most sensitive consumer group.

## 4. Conclusions

The results of this investigation showed that leafy vegetables had the highest frequency of MRL exceedances among the investigated vegetable groups. Several samples contained residues of multiple pesticides, in some cases up to ten different compounds. A total of 42 different pesticides, including their metabolites, were identified across the analyzed samples, confirming the widespread occurrence of multi-residue contamination.

Boscalid (32.3%), Azoxystrobin (21.0%), DDE p,p (19.7%), Acetamiprid (17.7%), Pyraclostrobin (17.7%), and Flonicamid (16.1%) were the most frequently occurring pesticides. Moderately hazardous pesticides constituted the largest portion of the detected residues, accounting for 36.6% of the total. Fungicides and insecticides were the most frequently identified classes of pesticides. Furthermore, the presence of pesticides not authorised for use as plant protection products, such as Acetochlor, Benalaxyl, Biphenyl, Chlorfenapyr, DDT, Dimethomorph, Diphenylamine, Fipronil, Imidacloprid, Linuron, and Metaflumizone, could suggest either regulatory non-compliance or environmental persistence.

This study demonstrates that the cultivation method and the type of vegetables can influence the levels of pesticide residue. Greenhouse-grown samples showed higher total pesticide residue concentrations and higher frequency of multiple residues compared with open-field samples. Moreover, leafy vegetables displayed higher residue concentrations than fruity vegetables indicating a more frequent accumulation of pesticides in this commodity group. Non-parametric statistical analysis supported these results by revealing that the differences observed are statistically significant. These findings suggest that microclimatic conditions in greenhouses, like temperature, humidity and limited ventilation, may contribute to increased pesticide residue accumulation. Therefore, improved greenhouse management practices, including optimized temperature regulation, humidity control, and ventilation, may help reduce residue levels and enhance compliance with food safety standards.

The estimated long-term dietary exposure represented only a small fraction of the acceptable daily intake (ADI), indicating a low chronic risk for consumers. However, the acute dietary exposure assessment revealed exceedances of the acute reference dose (ARfD) for several pesticide–vegetable combinations. In particular, children were identified as the most vulnerable consumer group, with ARfD exceedances observed for lettuce (Pyraclostrobin 369% and Tebuconazole 310%), spinach (Pyraclostrobin 118% and Tebuconazole 153%), and peppers (Acetamiprid 321%). For adults, exceedances were also observed for lettuce (Pyraclostrobin 118% and Tebuconazole nearly 99%). These findings emphasize that in Albania, there is a need for continuous surveillance and stricter management practices to minimize potential health risks associated with pesticide residues in vegetables.

## Figures and Tables

**Table 1:** Number of collected samples per commodity.

Leafy Vegetables		Fruity Vegetables		Root Vegetables		Bulb Vegetables	
Commodity	No. of Samples	Commodity	No. of Samples	Commodity	No. of Samples	Commodity	No. of Samples
Lettuce	12	Peppers	17	Potatoes	7	Onions	5
Spinach	10			Carrots	4	Leek (Allium porrum)	3
Parsley	2						
Dill	1						
Arugula	1						

This classification provided representative coverage of major vegetable categories and facilitated comparative analysis across distinct plant matrices. The collected samples were placed in labelled plastic containers and were immediately transferred to the laboratory for further processing.

**Table 2:** Leafy vegetables.

Vegetable	Sample No.	Open Field	Analyte	Value Found (mg/kg)	EU-MRL (mg/kg)
Lettuce	1	Open field	n.d ^1^		
	2	Open field	n.d		
	3	Open field	n.d		
	4	Greenhouse	Azoxystrobin	0.031	10
			Boscalid	0.13	50
			Chlorantraniliprole	0.014	20
			Difenoconazole	0.14	4
			Pyraclostrobin	0.017	2
	5	Open field	DDE-p,p’	Trace ^2^	0.05
	6	Greenhouse	n.d		
	7	Greenhouse	Azoxystrobin	0.45	10
			Boscalid	12.51	50
			Mandipropamid	0.55	25
			Metalaxyl	1.17	3
			Propamocarb	0.015	40
			**Pyraclostrobin**	**2.47**	**2**
			Tebuconazole	0.24	0.5
			Acetamiprid	0.034	1.5
	8	Greenhouse	Azoxystrobin	1.24	10
			Boscalid	1.41	50
			Metalaxyl	2.03	3
			**Pyraclostrobin**	**2.91**	**2**
			**Acetochlor**	**0.098**	**0.01**
			**Tefluthrin**	**0.019**	**0.01**
	9	Greenhouse	Acetamiprid	0.044	1.5
			Azoxystrobin	2.06	10
			Boscalid	0.03	50
			Mandipropamid	2.80	25
			Propamocarb	4.35	40
			**Tebuconazole**	**2.44**	**0.5**
	10	Greenhouse	**Fipronil**	**0.011**	**0.005**
			Mandipropamid	0.015	25
			Tebuconazole	0.2	0.5
	11	Greenhouse	Avermectin B1A	0.013	0.03
			Azoxystrobin	2.35	10
			Boscalid	6.08	50
			Metalaxyl	0.036	3
			Propamocarb	0.32	40
			Pyraclostrobin	1.07	2
	12	Greenhouse	Boscalid	0.017	50
			Mandipropamid	0.95	25
			Metalaxyl	1.04	3
			Spinosad	0.05	4
Spinach	1	Open field	n.d		
	2	Open field	Acetamiprid	0.136	0.6
			Boscalid	0.069	50
			Pyraclostrobin	0.03	0.6
	3	Open field	Acetamiprid	0.012	0.6
			DDTs ^3^	Trace	0.05
	4	Open field	Chlorantraniliprole	2.05	20
	5	Greenhouse	Acetamiprid	0.157	0.6
			**Avermectin B1A**	**0.015**	**0.01**
			Azoxystrobin	0.494	15
			Boscalid	0.048	50
			Chlorantraniliprole	0.033	20
	6	Greenhouse	Boscalid	0.022	50
			Chlorantraniliprole	0.18	20
	7	Greenhouse	Azoxystrobin	1.11	15
			Boscalid	0.863	50
			Fludioxonil	0.011	30
			**Pyraclostrobin**	**1.57**	**0.6**
			**Tebuconazole**	**2.03**	**0.02**
	8	Greenhouse	Azoxystrobin	0.26	15
			Boscalid	0.029	50
			**Fipronil**	**0.041**	**0.005**
			**Tebuconazole**	**0.23**	**0.02**
	9	Open field	Boscalid	0.011	50
	10	Greenhouse	Azoxystrobin	0.014	15
			Boscalid	0.015	50
			Mandipropamid	0.047	25
Parsley	1	Greenhouse	Azoxystrobin	1.77	70
			Boscalid	16.2	50
			Cyprodinil	0.088	40
			Fludioxonil	0.011	20
			Pendimethalin	0.017	2
			Propamocarb	0.011	30
			**Pyraclostraboin**	**3.73**	**2**
			**Tebuconazole**	**7.07**	**2**
			**2-phenylphenol**	**0.021**	**0.02**
			Biphenyl	0.026	0.1
	2	Greenhouse	Boscalid	0.13	50
			Pyraclostraboin	0.051	2
			**2-phenylphenol**	**0.046**	**0.02**
			Biphenyl	0.019	0.1
			**Tefluthrin**	**0.041**	**0.02**
Dill	1	Greenhouse	Biphenyl	0.02	0.05
			Pirimiphos-methyl	0.011	3
			**Azoxystrobin**	**1.37**	**0.3**
			**Boscalid**	**10.75**	**0.9**
			Cyprodinil	0.064	0.1
			Pendimethalin	0.04	0.05
			**Pyraclostrobin**	**2.49**	**0.1**
			**Tebuconazole**	**2.84**	**1.5**
Arugula	1	Greenhouse	Azoxystrobin	1.53	10
			Boscalid	12.74	50
			Pyraclostrobin	2.63	10
			**Tebuconazole**	**5.33**	**0.5**

^1^ n.d—No pesticide detected. ^2^ Trace indicates detectable residues below the limit of quantification (LOQ = 0.01 mg/kg). ^3^ DDTs include metabolites DDD-o,p, DDE-o,p, DDE-p,p’, and DDT-p,p’, detected at trace levels. Note: Analytes highlighted in bold and red indicate exceedance of MRLs.

**Table 3:** Fruity vegetables.

Vegetable	Sample No.	Cultivation Method	Analyte	Value Found (mg/kg)	EU-MRL (mg/kg)
Peppers	1	Greenhouse	Acetamiprid	0.023	0.3
			Flonicamid	0.061	0.3
			Avermectin B1a	Trace	0.03
	2	Greenhouse	Acetamiprid	0.013	0.3
			Flonicamid	0.095	0.3
			Mandipropamid	0.011	1
			Avermectin B1a	Trace	0.03
	3	Greenhouse	Flonicamid	0.062	0.3
	4	Greenhouse	Acetamiprid	0.071	0.3
			Difenoconazole	0.077	0.9
			Fenpyroximat	0.026	0.3
			Flonicamid	0.29	0.3
			Hexythiazox	0.01	0.09
			Tebuconazole	0.011	0.6
	5	Greenhouse	Acetamiprid	0.023	0.3
			Flonicamid	0.18	0.3
			Metaflumizone	0.073	1.5
			Pyriproxyfen	0.016	0.6
	6	Greenhouse	Acetamiprid	0.27	0.3
			**Fenazaquin**	**0.29**	**0.01**
			**Flonicamid**	**0.40**	**0.3**
			Imidacloprid	Trace	0.9
			Pyridaben	0.06	0.3
	7	Open field	**Benalaxyl**	**0.033**	**0.01**
			Pyriproxyfen	0.031	0.6
	8	Open field	n.d ^1^		
	9	Open field	Imidacloprid	Trace	0.9
	10	Open field	Tebufenpyrad	Trace	0.01
	11	Greenhouse	Flonicamid	0.04	0.3
			Pyrimethani	0.03	2
			**Tebufenpyrad**	**0.032**	**0.01**
	12	Open field	n.d		
	13	Open field	n.d		
	14	Greenhouse	Difenoconazole	0.13	0.9
			Fosthiazate	Trace	
			Chlorfenapyr	Trace	0.01
	15	Greenhouse	Difenoconazole	0.023	0.9
			Flonicamid	0.12	0.3
			**Chlorfenapyr**	**0.05**	**0.01**
	16	Greenhouse	Chlorantraniliprole	0.029	1
			Flonicamid	0.043	0.3
			Chlorfenapyr	0.012	0.01
	17	Greenhouse	Acetamiprid	0.026	0.3
			Boscalid	0.015	3
			Trifloxystrobin	0.012	0.9
			Flonicamid	0.091	0.3

^1^ n.d—No pesticide detected. Note: Analytes highlighted in bold and red indicate exceedance of MRLs.

**Table 4:** Root vegetables.

Vegetable	Sample No.	Cultivation Method	Analyte	Value Found (mg/kg)	EU-MRL (mg/kg)
Potatoes	1	Open field	n.d ^1^		
	2	Open field	Pirimiphos-methyl	Trace	0.01
			DDTs	Trace	0.05
	3	Open field	DDE-p,p’	Trace	0.05
	4	Open field	DDE-p,p’	Trace	0.05
	5	Open field	DDE-p,p’	Trace	0.05
	6	Open field	DDE-p,p’	Trace	0.05
	7	Open field	DDE-p,p’	Trace	0.05
			Tefluthrin	Trace	0.01
Carrots	1	Open field	n.d		
	2	Open field	Azoxystrobin	0.017	1
		Open field	Boscalid	0.013	2
		Open field	Tebuconazole	0.017	0.4
	3	Open field	n.d		
	4	Open field	Linuron	Trace	0.01
		Open field	DDE-p,p’	Trace	0.05

^1^ n.d—No pesticide detected.

**Table 5:** Bulb vegetables.

Vegetable	Sample No.	Cultivation Method	Analyte	Value Found (mg/kg)	EU-MRL (mg/kg)
Onions	1	Open field	Boscalid	0.03	5
			Chlorantraniliprole	0.012	0.01
			Cyprodinil	0.046	0.3
			Difenoconazole	0.011	0.5
			Dimethomorph	0.092	0.6
			Fludioxonil	0.014	0.5
			Mandipropamid	0.013	0.1
			Pyraclostrobin	0.032	1.5
			DDE-p,p’	Trace	0.05
			Pirimiphos-methyl	Trace	0.01
	2	Open field	n.d ^1^		
	3	Open field	n.d		
	4	Open field	DDE-p,p’	Trace	0.05
	5	Open field	Diphenylamnie	Trace	0.05
Leek (Allium porrum)	1	Open field	DDE,p,p’	Trace	0.05
			Diphenylamine	Trace	0.05
	2	Open field	Diphenylamine	Trace	0.05
	3	Open field	Diphenylamine	Trace	0.05

^1^ n.d—No pesticide detected.

**Table 6:** Detection frequency and levels of pesticide residues in vegetables.

Comodity	Analyte	Freq.	Mean Residues (mg/kg)	Max Residues (mg/kg)	Analyte	Freq.	Mean Residues (mg/kg)	Max Residues (mg/kg)
Lettuce	Acetamiprid	2	0.039	0.044	Fipronil	1	0.011	0.011
	Acetochlor	1	0.098	0.098	Mandipropamid	4	1.08	2.8
	Avermectin B1A	1	0.013	0.013	Metalaxyl	4	1.07	2.03
	Azoxystrobin	5	1.23	2.35	Propamocarb	3	1.56	4.35
	Boscalid	6	3.36	12.51	Pyraclostrobin	4	1.62	2.91
	Chlorantraniliprole	1	0.01	0.014	Spinosad	1	0.05	0.05
	DDE-p,p’	1	Trace	Trace	Tebuconazole	3	0.96	2.44
	Difenoconazole	1	0.14	0.14	Tefluthrin	1	0.019	0.019
Spinach	Acetamiprid	3	0.1	0.16	DDE-p,p’	1	Trace	Trace
	Avermectin B1A	1	0.015	0.015	DDT-p,p’	1	Trace	Trace
	Azoxystrobin	4	0.47	1.11	Fipronil	1	0.041	0.041
	Boscalid	7	0.151	0.863	Fludioxonil	1	0.011	0.011
	Chlorantraniliprole	3	0.75	2.05	Mandipropamid	1	0.047	0.047
	DDTs	1	Trace	Trace	Pyraclostrobin	2	0.8	1.57
	DDE-o,p	1	Trace	Trace	Tebuconazole	2	1.13	2.03
Parsley	2-phenylphenol	2	0.03	0.046	Pendimethalin	1	0.017	0.017
	Azoxystrobin	1	1.77	1.77	Propamocarb	1	0.011	0.011
	Biphenyl	2	0.02	0.026	Pyraclostrobin	2	1.89	3.73
	Boscalid	2	8.17	16.2	Tebuconazole	1	7.07	7.07
	Cyprodinil	1	0.09	0.088	Tefluthrin	1	0.041	0.041
	Fludioxonil	1	0.011	0.011				
Dill	Azoxystrobin	1	1.37	1.37	Pendimethalin	1	0.04	0.04
	Biphenyl	1	0.02	0.02	Pirimiphos-methyl	1	0.011	0.011
	Boscalid	1	10.75	10.75	Pyraclostrobin	1	2.49	2.49
	Cyprodinil	1	0.064	0.064	Tebuconazole	1	2.84	2.84
Arugula	Azoxystrobin	1	1.53	1.53	Pyraclostrobin	1	2.63	2.63
	Boscalid	1	12.74	12.74	Tebuconazole	1	5.33	5.33
Peppers	Acetamiprid	6	0.072	0.27	Hexythiazox	1	0.01	0.01
	Avermectin B1a	2	Trace	Trace	Imidacloprid	2	Trace	Trace
	Benalaxyl	1	0.033	0.033	Mandipropamid	1	0.011	0.011
	Boscalid	1	0.015	0.015	Metaflumizone	1	0.073	0.073
	Chlorantraniliprole	1	0.029	0.029	Pyridaben	1	0.06	0.06
	Chlorfenapyr	3	0.022	0.05	Pyrimethanil	1	0.025	0.025
	Difenoconazole	3	0.078	0.13	Pyriproxyfen	2	0.024	0.031
	Fenazaquin	1	0.29	0.29	Tebuconazole	1	0.011	0.011
	Fenpyroximat	1	0.026	0.026	Tebufenpyrad	2	0.02	0.03
	Flonicamid	10	0.15	0.4	Trifloxystrobin	1	0.012	0.012
	Fosthiazate	1	Trace	Trace				
Potatoes	DDE-o,p’	1	Trace	Trace	Pirimiphos-methyl	1	Trace	Trace
	DDE-p,p’	6	Trace	Trace	Tefluthrin	1	Trace	Trace
	DDT-p, p’	1	Trace	Trace				
Carrots	Azoxystrobin	1	0.017	0.017	Linuron	1	Trace	Trace
	Boscalid	1	0.013	0.013	Tebuconazole	1	0.017	0.017
	DDE-p,p’	1	Trace	Trace				
Onions	Boscalid	1	0.03	0.03	Diphenylamnie	1	Trace	Trace
	Chlorantraniliprole	1	0.012	0.012	Fludioxonil	1	0.014	0.014
	Cyprodinil	1	0.046	0.046	Mandipropamid	1	0.013	0.013
	DDE-p,p’	2	Trace	Trace	Pirimiphos-methyl	1	Trace	Trace
	Difenoconazole	1	0.011	0.011	Pyraclostrobin	1	0.032	0.032
	Dimethomorph	1	0.092	0.092				
Leek (Allium porrum)	DDE,p,p’	1	Trace	Trace	Diphenylamine	3	Trace	Trace
	DDE,p,p’	1	Trace	Trace	Diphenylamine	3	Trace	Trace

**Table 7:** Categorization of detected pesticides in vegetables.

No.	Pesticide	Chemical Class	Main Use	Hazard Classification *
1	2-phenylphenol	Aromatic phenols	Fungicide	Slight
2	Acetamiprid	Neonicotinoid	Insecticide	Moderate
3	Acetochlor	Chloroacetanilide	Herbicide	Slight
4	Avermectin B1a	Macrocyclic Lactone	Insecticide	High
5	Azoxystrobin	Strobin	Fungicide	U **
6	Benalaxyl	Xylylalanine	Fungicide	Slight
7	Biphenyl	Unclassified	Fungicide, Microbiocide	Slight
8	Boscalid	Anilide	Fungicide	U
9	Chlorantraniliprole	Anthranilic diamide	Insecticide	U
10	Chlorfenapyr	Pyrazole	Insecticide	Moderate
11	Cyprodinil	Pyrimidine	Fungicide	Insufficiently Studies
12	DDT and metabolites	Organochlorine	Insecticide	Moderate
16	Difenoconazole	Azole	Fungicide	Moderate
17	Dimethomorph	Morpholine	Fungicide	Slight
18	Diphenylamnie	Amine	Fungicide, Insecticide, Plant Growth Regulator	Insufficiently Studies
19	Fenazaquin	No Data	Insecticide	Moderate
20	Fenpyroximat	Pyrazole	Insecticide	Moderate
21	Fipronil	Pyrazole	Insecticide	Moderate
22	Flonicamid	Unclassified	Insecticide	Moderate
23	Fludioxonil	Pyrrole	Fungicide	U
24	Fosthiazate	Organophosphorus	Nematicide	High
25	Hexythiazox	Unclassified	Plant Growth Regulator	U
26	Imidacloprid	Neonicotinoid	Insecticide	Moderate
27	Linuron	Urea	Herbicide	Slight
28	Mandipropamid	No Data	Fungicide	U
29	Metaflumizone	Unclassified	Insecticide	U
30	Metalaxyl	Xylylalanine	Fungicide	Moderate
31	Pendimethalin	2,6-Dinitroaniline	Herbicide	Moderate
32	Pirimiphos-methyl	Organophosphorus	Insecticide	Moderate
33	Propamocarb	Other Carbamate	Fungicide	U
34	Pyraclostrobin	Strobin	Fungicide	Slight
35	Pyridaben	Unclassified	Insecticide	Moderate
36	Pyriproxyfen	Juvenile hormone mimic	Plant Growth Regulator	U
37	Pyrimethani	Anilinopyrimidines	Fungicide	Slight
38	Spinosad	Spinosyn, Macrocyclic Lactone	Insecticide	Slight
39	Tebuconazole	Azole	Fungicide	Moderate
40	Tebufenpyrad	Pyrazole	Insecticide	Moderate
41	Tefluthrin	Pyrethroid	Insecticide	High
42	Trifloxystrobin	Strobin	Fungicide	U

* The WHO Recommended Classification of Pesticides by Hazard. U ** unlikely to present acute hazard in normal use.

**Table 8:** Impact of cultivation system and microclimatic conditions on pesticide residues.

Vegetables	District	Greenhouse			Open Field		
		Exceeding MRL	Below MRL	Pesticide Free	Exceeding MRL	Below MRL	Pesticide Free
Leafy	Tirane	11	2	0	0	2	0
	Durres	0	1	0	0	0	0
	Elbasan	0	0	0	0	1	0
	Berat	0	0	1	0	0	2
	Fier	0	1	0	0	1	0
	Korca	0	0	0	0	2	2
Fruity	Shkoder	1				1	1
	Puke						1
	Kukes	0	0	0	0	1	0
	Tirane		1		1		1
	Berat	0	4	0	0	0	0
	Fier	1	1				
	Korca	1	2	0	0	0	0
% of samples		50	42.9	3.6	6.3	50	43.8

**Table 9:** Long-Term Dietary Exposure Expressed as %ADI for Detected Pesticide Residues.

Comodity	Analyte	Mean Residues (mg/kg)	ADI (mg/kg bw/day)	%ADI (Toddler)	%ADI (Adult)
Lettuce	Acetamiprid	0.039	0.005	0.23	0.29
	Acetochlor	0.098	0.0036	0.79	1.00
	Avermectin b1A	0.013	0.0012	0.32	0.41
	Azoxystrobin	1.230	0.2	0.18	0.23
	Boscalid	3.360	0.04	2.00	3.00
	Chlorantraniliprole	0.010	1.56	0.00	0.00
	Difenoconazole	0.140	0.01	0.41	0.53
	Fipronil	0.011	0.0002	2.00	2.00
	Mandipropamid	1.080	0.15	0.21	0.27
	Metalaxyl	1.070	0.08	0.39	0.50
	Propamocarb	1.560	0.29	0.16	0.20
	Pyraclostrobin	1.620	0.03	2.00	2.00
	Spinosad	0.050	0.024	0.06	0.08
	Tebuconazole	0.960	0.03	0.93	1.00
	Tefluthrin	0.019	0.005	0.11	0.14
Spinach	Acetamiprid	0.100	0.005	0.12	0.19
	Avermectin b1A	0.015	0.0012	0.07	0.12
	Azoxystrobin	0.470	0.2	0.01	0.02
	Boscalid	0.150	0.04	0.02	0.04
	Chlorantraniliprole	0.750	1.56	0.00	0.00
	Fipronil	0.041	0.0002	1.00	2.00
	Fludioxonil	0.011	0.37	0.00	0.00
	Mandipropamid	0.047	0.15	0.00	0.00
	Pyraclostrobin	0.800	0.03	0.16	0.25
	Tebuconazole	1.130	0.03	0.22	0.36
Parsley	2-phenylphenol	0.030	0.4	0.00	0.00
	Azoxystrobin	1.770	0.2	0.01	0.01
	Biphenyl	0.020	0.038	0.00	0.00
	Boscalid	8.170	0.04	0.18	0.21
	Cyprodinil	0.090	0.03	0.00	0.00
	Fludioxonil	0.011	0.37	0.00	0.00
	Pendimethalin	0.017	0.125	0.00	0.00
	Propamocarb	0.011	0.29	0.00	0.00
	Pyraclostrobin	1.890	0.03	0.06	0.06
	Tebuconazole	7.070	0.03	0.21	0.24
	Tefluthrin	0.041	0.005	0.01	0.01
Peppers	Acetamiprid	0.072	0.005	0.09	0.10
	Benalaxyl	0.033	0.04	0.01	0.01
	Boscalid	0.015	0.04	0.00	0.00
	Chlorantraniliprole	0.029	1.56	0.00	0.00
	Chlorfenapyr	0.022	0.015	0.01	0.01
	Difenoconazole	0.078	0.01	0.05	0.05
	Fenazaquin	0.290	0.005	0.35	0.40
	Fenpyroximat	0.026	0.01	0.02	0.02
	Flonicamid	0.150	0.025	0.04	0.04
	Hexythiazox	0.010	0.03	0.00	0.00
	Mandipropamid	0.011	0.15	0.00	0.00
	Metaflumizone	0.073	0.01	0.04	0.05
	Pyridaben	0.060	0.01	0.04	0.04
	Pyrimethanil	0.025	0.17	0.00	0.00
	Pyriproxyfen	0.024	0.05	0.00	0.00
	Tebuconazole	0.011	0.03	0.00	0.00
	Tebufenpyrad	0.020	0.01	0.01	0.01
	Trifloxystrobin	0.012	0.1	0.00	0.00
Carrots	Azoxystrobin	0.017	0.2	0.00	0.00
	Boscalid	0.013	0.04	0.00	0.00
	Tebuconazole	0.017	0.03	0.01	0.01
Onions	Boscalid	0.030	0.04	0.01	0.01
	Chlorantraniliprole	0.012	1.56	0.00	0.00
	Cyprodinil	0.046	0.03	0.02	0.02
	Difenoconazole	0.011	0.01	0.01	0.01
	Dimethomorph	0.092	0.05	0.02	0.02
	Fludioxonil	0.014	0.37	0.00	0.00
	Mandipropamid	0.013	0.15	0.00	0.00
	Pyraclostrobin	0.032	0.03	0.01	0.01

**Table 10:** Short-Term Dietary Exposure Expressed as % ARfD for children and adults.

Comodity	Analyte	Highest Residue (mg/kg)	ARfD (mg/kg bw)	% ARfD (Children)	% ARfD (Adult)
Lettuce	Acetamiprid	0.044	0.005	34.0	11.0
	Acetochlor	0.098	1.5	0.2	0.08
	Avermectin b1A	0.013	0.0012	41.0	13.0
	Difenoconazole	0.140	0.16	3.0	1.0
	Fipronil	0.011	0.009	5.0	1.0
	Metalaxyl	2.030	0.5	15.0	5.0
	Propamocarb	4.350	1	17.0	5.0
	Pyraclostrobin	2.910	0.03	**369.0**	**118.0**
	Spinosad	0.050	0.1	2.0	0.6
	Tebuconazole	2.440	0.03	**310.0**	**99.0**
	Tefluthrin	0.019	0.005	14.0	5.0
Spinach	Acetamiprid	0.160	0.005	72.0	13.0
	Avermectin b1A	0.015	0.0012	28.0	5.0
	Fipronil	0.041	0.009	10.0	2.0
	Pyraclostrobin	1.570	0.03	**118.0**	21.0
	Tebuconazole	2.030	0.03	**153.0**	27.0
Parsley	Pendimethalin	0.017	0.3	0.0062	0.01
	Propamocarb	0.011	1	0.0012	0.0
	Pyraclostrobin	3.730	0.03	14.0	15.0
	Tebuconazole	7.070	0.03	26.0	28.0
	Tefluthrin	0.041	0.005	0.9	1.0
Peppers	Acetamiprid	0.270	0.005	**321.0**	88.0
	Benalaxyl	0.033	0.5	0.4	0.1
	Chlorfenapyr	0.050	0.015	20.0	5.0
	Difenoconazole	0.130	0.16	5.0	1.0
	Fenazaquin	0.290	0.1	15.0	4.0
	Fenpyroximat	0.026	0.02	8.0	2.0
	Flonicamid	0.400	0.025	95.0	26.0
	Metaflumizone	0.073	0.13	3.0	0.9
	Pyridaben	0.060	0.05	7.0	2.0
	Pyriproxyfen	0.031	1	0.2	0.05
	Tebuconazole	0.011	0.03	2.0	0.6
	Tebufenpyrad	0.030	0.02	9.0	2.0
	Trifloxystrobin	0.012	0.5	0.1	0.04
Carrots	Tebuconazole	0.017	0.03	4.0	1.0
Onions	Difenoconazole	0.011	0.16	0.2	0.1
	Dimethomorph	0.092	0.6	0.3	0.2
	Pyraclostrobin	0.032	0.03	2.0	2.0

Values highlighted in bold and red indicate exceedance of short-term dietary exposure.

## Data Availability

The original contributions presented in this study are included in the article/Supplementary Material. Further inquiries can be directed to the corresponding author.

## Supplementary Materials

The following supporting information can be downloaded at: https://www.mdpi.com/article/10.3390/foods15101761/s1, Tables S1 and S2: Detailed analytical parameters for all target pesticides analysed in this study, including compound names, precursor and product ions, retention times, and collision energies for LC-MS/MS (Table S1) and GC-MS/MS (Table S2). Table S3: Detailed information about the sampling framework, including region, cultivation method, and planting/harvesting schedules. Tables S4 and S5: Detailed information about method validation according to SANTE guidelines. Table S6: Statistical analysis of pesticide residues between greenhouse and open-field samples.

## Abbreviations

The following abbreviations are used in this manuscript:

GC-MS/MS	gas chromatography–tandem mass spectrometry
LC-MS/MS	liquid chromatography–tandem mass spectrometry
MRL	maximum residue limit
QuEChERS	Quick, Easy, Cheap, Effective, Rugged, Safe
ESI	An electrospray ionisation
EI	electron impact
MRM	multiple reactions monitoring
LOD	limit of detection
LOQ	limit of quantification
PRIMo	Pesticide Residue Intake Model
STMR	Supervised Trials Median Residue
HR	Highest Residue
IESTI	Estimated Short-Term Intake
ARfD	Acute Reference Dose
NEDI	National Estimated Daily Intake
ADI	Acceptable Daily Intake

## References

[1] Herforth A., Arimond M., Álvarez-Sánchez C., Coates J., Christianson K., Muehlhoff E. (2019). A Global Review of Food-Based Dietary Guidelines. Adv. Nutr..

[2] Devirgiliis C., Guberti E., Mistura L., Raffo A. (2024). Effect of Fruit and Vegetable Consumption on Human Health: An Update of the Literature. Foods.

[3] Schwingshackl L., Morze J., Hoffmann G. (2020). Mediterranean Diet and Health Status: Active Ingredients and Pharmacological Mechanisms. Br. J. Pharmacol..

[4] de Oliveira Elias S., Noronha T.B., Tondo E.C. (2019). *Salmonella* spp. and *Escherichia coli* O157:H7 Prevalence and Levels on Lettuce: A Systematic Review and Meta-Analysis. Food Microbiol..

[5] Legal Framework—IPARD 2014–2020. https://ipard.gov.al/legal-framework/.

[6] Statistikat e Bujqësisë, 2024. Instat. https://www.instat.gov.al/sq/temat/bujqesia-dhe-peshkimi/bujqesia/publikimet/2025/statistikat-e-bujqesise-2024/.

[7] Jankowska M., Kaczyński P., Hrynko I., Rutkowska E., Iwaniuk P., Ilyasova G., Łozowicka B. (2024). Dietary Risk Assessment of Children and Adults Consuming Fruit and Vegetables with Multiple Pesticide Residues. Chemosphere.

[8] Wang J., Chow W., Chang J., Wong J.W. (2017). Development and Validation of a Qualitative Method for Target Screening of 448 Pesticide Residues in Fruits and Vegetables Using UHPLC/ESI Q-Orbitrap Based on Data-Independent Acquisition and Compound Database. J. Agric. Food Chem..

[9] Baudry J., Rebouillat P., Samieri C., Berlivet J., Kesse-Guyot E. (2023). Dietary pesticide exposure and non-communicable diseases and mortality: A systematic review of prospective studies among adults. Environ. Health.

[10] Chmielewski J.P., Wszelaczyńska E., Pobereżny J., Gworek B., Walosik A., Florek-Łuszczki M. (2025). Effect of consumption of vegetables contaminated with pesticides on consumers’ health—Risk analysis. Ann. Agric. Env. Med..

[11] Authority E.F.S., Carrasco Cabrera L., Di Piazza G., Dujardin B., Marchese E., Medina Pastor P. (2025). The 2023 European Union Report on Pesticide Residues in Food. EFSA J..

[12] Baša Česnik H., Velikonja Bolta Š. (2024). Pesticide residues in vegetables: Validation of the gas chromatography–tandem mass spectrometry multiresidual method and a survey of vegetables on the Slovenian market. Acta Agric. Slov..

[13] Łozowicka B., Kaczyński P., Jankowska M., Rutkowska E., Iwaniuk P., Konecki R., Rogowska W., Zhagyparova A., Absatarova D., Łuniewski S. (2025). Evaluation of broad-spectrum pesticides based on unified multi-analytical procedure in fruits and vegetables for acute health risk assessment. Foods.

[14] Rutkowska E., Kaczyński P., Iwaniuk P., Łozowicka B., Hrynko I., Jankowska M., Konecki R., Rogowska W., Rusiłowska J., Pietkun M. (2025). An extensive pesticide residue study in minor Polish vegetables based on critical consumer diets. Food Control.

[15] Orecchio S., Arrabito G.D., Amorello D., Di Gaudio F., Barreca S., Orecchio S. (2026). Quantification of pesticide residues in fruits and vegetables sampled in Sicily (Italy) and assessment of health risks. J. Food Compos. Anal..

[16] Atzei A., Bouakline H., Corrias F., Angioni A. (2025). Four-year monitoring survey of pesticide residues in tomato samples: Human health and environmental risk assessment. J. Xenobiotics.

[17] (2005). Regulation (EC) No 396/2005 of the European Parliament and of the Council of 23 February 2005 on Maximum Residue Levels of Pesticides in or on Food and Feed of Plant and Animal Origin and Amending Council Directive 91/414/EEC Text with EEA Relevance. http://data.europa.eu/eli/reg/2005/396/oj.

[18] Branković M. (2026). Pesticide residues in food from Balkan countries exported to the European Union. Food Control.

[19] AKU Bllokon 20-ton Produkt Kastravec. Autoriteti Kombëtar i Ushqimit. https://aku.gov.al/2023/11/20/aku-bllokon-20-ton-produkt-kastravec/.

[20] Informacion për Publikun. Autoriteti Kombëtar i Ushqimit. https://aku.gov.al/2024/12/23/informacion-per-publikun-4/.

[21] Likaj M., Marku E., Mediu R., Tahiraj J., Shehu S. Comprehensive Evaluation of Pesticide Residues in Albanian Tomatoes by Advanced Chromatographic and Mass Spectrometric Techniques. Proceedings of the UBT International Conference.

[22] Likaj M., Marku E., Mediu R., Tahiraj J., Shehu S. (2025). Preliminary Study on Pesticide Contamination in Albanian Agricultural Soils: Challenges, Risk, and Sustainable Management Strategies. J. Hyg. Eng. Des..

[23] Anastassiades M., Lehotay S., štajnbaher D., Schenck F. (2003). Fast and Easy Multiresidue Method Employing Acetonitrile Extraction/Partitioning and “Dispersive Solid-Phase Extraction” for the Determination of Pesticide Residues in Produce. J. AOAC Int..

[24] (2002). Commission Directive 2002/63/EC of 11 July 2002 Establishing Community Methods of Sampling for the Official Control of Pesticide Residues in and on Products of Plant and Animal Origin and Repealing Directive 79/700/EEC. http://data.europa.eu/eli/dir/2002/63/oj.

[25] (2018). Pesticide Residue Analysis in Plant Foods QuEChERS Method.

[26] European Commission Directorate-General for Health and Food Safety (SANTE/11312/2021) EURL|Residues of Pesticides|Analytical Quality Control and Method Validation Procedures for Pesticide Residues Analysis in Food and Feed. https://www.eurl-pesticides.eu/docs/public/tmplt_article.asp?CntID=727.

[27] European Commission Directorate-General for Health and Food Safety (2026). SANTE/11312/2021 V2026—Guidance Document on Analytical Quality Control and Method Validation Procedures for Pesticide Residues Analysis in Food and Feed.

[28] (2017). General Requirements for the Competence of Testing and Calibration Laboratories.

[29] European Food Safety Authority (EFSA) (2018). Pesticide Residue Intake Model (PRIMo), Revision 3.1. EFSA Pesticide Evaluation: Tools. https://www.efsa.europa.eu/en/applications/pesticides/tools.

[30] De Rosa E., Di Lillo M., Triassi M., Di Duca F., Russo I., Graziano V., Mazzei G., Gentile I., Shojaeian S.Z., Montuori P. (2025). Analysis and Risk Assessment of Pesticide Residues in Strawberry Using the PRIMo Model: Detection, Public Health and Safety Implications. Foods.

[31] European Food Safety Authority (EFSA) (2011). Use of the EFSA Comprehensive European Food Consumption Database in exposure assessment. EFSA J..

[32] EFSA Scientific Committee (2018). Guidance on uncertainty analysis in scientific assessments. EFSA J..

[33] Brancato A., Brocca D., Ferreira L., Greco L., Jarrah S., Leuschner R., Medina P., Miron I., Nougadère A., Pedersen R. (2018). Use of EFSA Pesticide Residue Intake Model (EFSA PRIMo Revision 3). EFSA J..

[34] El-Sheikh E.-S.A., Ramadan M.M., El-Sobki A.E., Shalaby A.A., McCoy M.R., Hamed I.A., Ashour M.-B., Hammock B.D. (2022). Pesticide Residues in Vegetables and Fruits from Farmer Markets and Associated Dietary Risks. Molecules.

[35] Zhang Y., Si W., Chen L., Shen G., Bai B., Zhou C. (2021). Determination and Dietary Risk Assessment of 284 Pesticide Residues in Local Fruit Cultivars in Shanghai, China. Sci. Rep..

[36] El-Sheikh E.-S.A., Li D., Hamed I., Ashour M.-B., Hammock B.D. (2023). Residue Analysis and Risk Exposure Assessment of Multiple Pesticides in Tomato and Strawberry and Their Products from Markets. Foods.

[37] Acosta-Dacal A., Díaz-Díaz R., Alonso-González P., Bernal-Suárez M.d.M., Parga-Dans E., Serra-Majem L., Ortiz-Andrellucchi A., Zumbado M., Santos E., Furtado V. (2025). Pesticide residues in fruits and vegetables from Cape Verde: A multi-year monitoring and dietary risk assessment study. Foods.

[38] Della Rovere I., Zianni R., Casamassima F.P., Accettulli A.M., Calitri A., Catano F., Nardelli V. (2026). Comprehensive assessment of pesticide residues in fruits and vegetables from Apulia and Basilicata (Southern Italy, 2022–2025) and related risk evaluation. Appl. Sci..

[39] Bucheli T.D., Barmettler E., Bartoloméa N., Hilber I., Hornak K., Meuli R.G., Reininger V., Riedo J., Rösch A., Sutter P. (2023). Pesticides in agricultural soils: Major findings from various monitoring campaigns in Switzerland. Chimia.

[40] Chemisches und Veterinäruntersuchungsamt Stuttgart (CVUA) (2021). Residues and Contaminants in Fresh Vegetables.

[41] Louca Christodoulou D., Kourouzidou O., Hadjigeorgiou M., Hadjiloizou P., Constantinou M., Constantinou P., Kika K., Klavarioti M. (2018). Multi-residue analysis of pesticide residues in fruits and vegetables using gas and liquid chromatography with mass spectrometric detection. Accredit. Qual. Assur..

[42] (2020). WHO Recommended Classification of Pesticides by Hazard and Guidelines to Classification.

